# Enhancer of zeste homolog 1/2 dual inhibitor valemetostat outperforms enhancer of zeste homolog 2-selective inhibitors in reactivating latent HIV-1 reservoirs *ex vivo*

**DOI:** 10.3389/fmicb.2025.1581330

**Published:** 2025-04-10

**Authors:** Ayako Sedohara, Tomohiko Koibuchi, Makoto Yamagishi, Michiko Koga, Kotaro Arizono, Kazuhiko Ikeuchi, Tadashi Kikuchi, Makoto Saito, Eisuke Adachi, Takeya Tsutsumi, Daisuke Honma, Kazushi Araki, Kaoru Uchimaru, Hiroshi Yotsuyanagi

**Affiliations:** 1 Division of Infectious Diseases, Advanced Clinical Research Center, Institute of Medical Science, The University of Tokyo, Minato-ku, Tokyo, Japan; 2 Department of Infectious Disease and Applied Immunology, IMSUT Hospital of The Institute of Medical Science, The University of Tokyo, Minato-ku, Tokyo, Japan; 3 Department of Computational Biology and Medical Sciences, Graduate School of Frontier Sciences, The University of Tokyo, Bunkyo-ku, Tokyo, Japan; 4 Department of Infectious Diseases, Faculty of Medicine, The University of Tokyo, Bunkyo-ku, Tokyo, Japan; 5 Modality Research Laboratories III, Daiichi Sankyo Co., Ltd., Shinagawa-ku, Tokyo, Japan; 6 Early Clinical Development Department, Daiichi Sankyo Co., Ltd., Shinagawa-ku, Tokyo, Japan

**Keywords:** HIV-1 reservoir, latency reversing agents, EZH1/2 dual inhibitor, valemetostat, suberoylanilide hydroxamic acid, GSK126, E7438

## Abstract

For the eradication of human immunodeficiency virus type 1 (HIV-1) provirus from people living with HIV-1, reactivation of latently HIV-1-infected cells is essential. Although several latency reversing agents have been identified, eradication of HIV-infected cells has been a challenge. Here, we investigated whether the novel enhancer of zeste homolog 1/2 (EZH1/2) dual inhibitor valemetostat/DS-3201/(*R*)-OR-S2 could efficiently reactivate latently HIV-1-infected cells *in vitro* and *ex vivo*. People living with HIV-1 who were on suppressive combined antiretroviral therapy and with plasma HIV-1 virus levels consistently below 50 copies/mL were enrolled in this study. ACH2 cells were treated with valemetostat for 7–14 days and with suberoylanilide hydroxamic acid (SAHA). CD4^+^ T cells were treated with valemetostat or the EZH2-selective inhibitors GSK126 and E7438 for 22 days alone or in combination with SAHA. *HIV-1* expression in CD4^+^ T cells was determined. Valemetostat more effectively induced *HIV-1* mRNA expression in ACH-2 cells when administered for 14 days than when administered for 7 days. Valemetostat reversed latently HIV-l-infected CD4^+^ T cells isolated from patients with HIV-1 and induced *HIV-1* mRNA expression more potently than GSK126 and E7438. In addition, valemetostat induced *HIV-1* mRNA expression more strongly when used in combination with SAHA compared with GSK126 and E7438. Expression levels of 21 hub genes were markedly increased after treatment with valemetostat. Gene Ontology analysis revealed that proteins encoded by these 21 genes were localized to the cell membrane and involved in the immune response. Kyoto Encyclopedia of Genes and Genomes enrichment pathway analysis showed that these 21 hub genes contributed to various signaling pathways, including the JAK–STAT signaling pathway. This study provides novel insights for the development of treatments to reactivate latently HIV-1-infected cells.

## 1 Introduction

Human immunodeficiency virus-1 (HIV-1) is an RNA virus targeting T CD4^+^ lymphocytes. The HIV-1 genome is converted into HIV-1 DNA fragments by reverse transcriptase, and these fragments, referred to as proviruses, are subsequently integrated into the host genomic DNA (Sadowski and Hashemi, 2019; Shan et al., 2017). Antiretroviral therapy prevents viral replication and new infections of CD4^+^ T cells; however, it cannot eliminate HIV-1-infected cells. CD4^+^ T cells possess self-renewal capability and have a long lifespan (Chahroudi et al., 2015). They carry the HIV-1 provirus over an extended period, complicating the eradication of HIV-1-infected cells (Chomont et al., 2009; Hosmane et al., 2017; Kwon et al., 2020). In addition, because the HIV-1 provirus is silenced by epigenetic modifications (Sadowski and Hashemi, 2019), infected CD4^+^ T cells do not express the HIV-1 antigen on the cell surface and evade cell-mediated immunity. These features render CD4^+^ T cells as latent HIV-1 reservoirs, the eradication of which in patients with HIV-1 infection is a crucial and long-standing therapeutic challenge.

The “kick and kill” strategy aims to eradicate latently HIV-1-infected CD4^+^ T cells by reactivating them with a latency reversing agent (LRA) and eliminating them by the host immune system activation (Sadowski and Hashemi, 2019; Thorlund et al., 2017). Various LRAs have been identified but eliminating HIV-infected cells *in vivo* remains challenging (Ait-Ammar et al., 2019; Gay et al., 2024; Kim et al., 2018; Ta et al., 2022). Enhancer of zeste homolog 2 (EZH2), a component of polycomb repressive complex 2, is a histone methyltransferase that methylates H3K27 (Holoch and Margueron, 2017). EZH2 contributes to chromatin compaction and prevents RNA polymerase II elongation, limiting access of both transcription factors and ATP-dependent chromatin-remodeling machinery to chromatin (Di Croce and Helin, 2013; Khan et al., 2018). EZH2 is involved in the silencing of intracellular *HIV-1* expression and maintenance of HIV-1 proviral latency (Friedman et al., 2011; Matsuda et al., 2015; Nguyen et al., 2017; Sharma et al., 2020). Therefore, EZH2-selective inhibitors reactivate intracellular *HIV-1* expression (Friedman et al., 2011; Matsuda et al., 2015; Nguyen et al., 2017; Turner et al., 2020). EZH1, an EZH2 homolog, also functions as a catalytic domain of PRC2 methyltransferase and compensates for the loss of EZH2 activity (Shen et al., 2008). To effectively suppress PRC2 methyltransferase activity, EZH1 and EZH2 should be inhibited simultaneously.

In this study, we investigated whether the novel EZH1/2 dual inhibitor valemetostat/DS-3201/(*R*)-OR-S2 (Honma et al., 2017; Yamagishi et al., 2019), which was approved in Japan for the treatment of relapsed or refractory adult T-cell leukemia/lymphoma in September 2022 (Izutsu et al., 2023; Keam, 2022) and peripheral T-cell lymphoma in June 2024 (Zinzani et al., 2024), could reactivate latently HIV-1-infected cells *in vitro* and *ex vivo* more effectively than the EZH2-selective inhibitors GSK126 (Gulati et al., 2018) and E7438 (Izutsu et al., 2021; Morschhauser et al., 2020). Furthermore, we used transcriptome analysis to compare differences in gene expression changes in CD4^+^ T cells treated with valemetostat, GSK126, and E7438.

## 2 Materials and methods

### 2.1 Patients and clinical samples

Patients infected with HIV-1 were enrolled in this study if they were on suppressive combined antiretroviral therapy for >3 years and with plasma HIV-1 virus levels consistently below 50 copies/mL for at least 2 years (Table 1). This study was reviewed and approved by the Research Ethics Committee of the University of Tokyo (approval No. 30-96-B20190402). All participants provided written informed consent to participate in the study and for their data to be published.

**Table 1:** Clinical characteristics of the study participants.

Subject	Current CD4^+^ T cell count (cells/μL)	Nadir CD4^+^ T cell count (cells/μL)	HIV-1 RNA (copies/mL)	Duration of the suppressing regimen (years)	ART regimen
P2-3	827	223	<50	10	TDF/FTC/RPV
P4-2/P4-3	886	164	<50	9	TAF/FTC, DTG
P5-3/P5-4	333	190	<50	10	TAF/FTC, DRV/cobi
P6-2/P6-3	1,176	259	<50	9	TAF/FTC, RAL
P7	574	150	<50	8	ABC/3TC, DRV/cobi
P8	797	250	<50	13	TAF/FTC, DTG
P10	733	45	<50	8	ABC/3TC/DTG
P11	252	73	<50	12	ABC/3TC/DTG
P12	1,254	169	<50	9	ABC/3TC/DTG
P13	227	73	<50	5	TAF/FTC, DTG
P14	632	222	<50	6	ABC/3TC/DTG
P16/P16-2	592	55	<50	18	TAF/FTC, DTG

ABC: abacavir; DTG: dolutegravir; DRV/cobi: draunavir boosted with cobicistat; FTC: emtricitabine; RPV: rilpivarine; TAF: tenofovir alafenamide; TDF: tenofovir; RAL: raltegravir; 3TC: lamivudine.

### 2.2 ACH2 cell culture

ACH2 cells (1.0 × 10^6^ cells) were cultured at 37°C in the atmosphere of 95% air and 5% CO_2_ in 12-well plates (Sumitomo Bakelite Co. Ltd., Tokyo, Japan) with 1 mL of Roswell Park Memorial Institute (RPMI) 1,640 medium (plus L-glutamine, without phenol red) (Gibco, New York, NY, USA) supplemented with 10% 0.22 μM filtered fetal bovine serum (Thermo Fisher Scientific, Waltham, MA, USA) and 0.6% of penicillin–streptomycin solution (Merck, Darmstadt, Germany).

### 2.3 Long-term primary CD4^+^ T cell culture

Peripheral blood mononuclear cells were isolated using SepMate™ (STEMCELL Technologies, Vancouver, BC, Canada) according to the manufacturer’s protocol. CD4^+^ T cells were isolated by negative selection using an EasySep™ Human CD4^+^ T Cell Isolation Kit (STEMCELL Technologies) and cultured at 37°C in the atmosphere of 95% air and 5% CO_2_ in a 48-well flat-bottomed plate (Sumitomo Bakelite Co.) filled with 500 μL of RPMI-1640 medium (plus L-glutamine, without phenol red; Gibco) supplemented with 10% 0.22 μM filtered fetal bovine serum (Thermo Fisher Scientific), 0.6% of penicillin–streptomycin solution (Merck), and 300 nM efavirenz (Merck), which prevented new HIV-1 infection. To maintain primary CD4^+^ T cells *ex vivo* for >7 days, they were treated with beads coated with antibodies against CD3 and CD28 (STEMCELL Technologies) for 4 days (Kuzmichev et al., 2017). After the removal of the beads, 5 ng/mL interleukin 7 (IL-7) was added every 3–4 days (Murera et al., 2018). Half of the culture medium was renewed every 7 days.

### 2.4 Inhibitor treatment

EZH2-selective inhibitors E7438 and GSK126 (Duan et al., 2020), and EZH1/2 dual inhibitor valemetostat/DS-3201/(*R*)-OR-S2 were synthesized in-house (Honma et al., 2017). Histone deacetylase inhibitor vorinostat/suberoylanilide hydroxamic acid (SAHA) (Merck) and reverse transcriptase inhibitor efavirenz (Merck) were dissolved in dimethyl sulfoxide (FUJIFILM Wako Pure Chemical Corporation, Osaka, Japan). Recombinant human IL-7 (Thermo Fisher Scientific) was dissolved in phosphate-buffered saline containing 0.1% bovine serum albumin (FUJIFILM Wako Pure Chemical Corporation).

ACH2 cells (1.0 × 10^6^) were treated with 100 nM valemetostat for 7–14 days and with the HDAC inhibitor SAHA (100 nM, 500 nM, or 1 μM), an LRA, for 24 h, 1 day before the last day of valemetostat treatment. CD4^+^ T cells (1.0 × 10^6^) were treated with 1,000 nM valemetostat, GSK126, or E7438 for 22 days alone or in combination with 500 nM SAHA for 6 h on the final day of treatment.

### 2.5 Quantitative gene expression analysis

Total RNA and genomic DNA were extracted from cells using ISOGEN II and ISOGENOME (Nippon Gene Co. Ltd., Tokyo, Japan), respectively. The total RNA precipitate was then dissolved in ddH_2_O. The DNA pellet was resuspended in 8 mM NaOH and adjusted to pH 8.0 with a 0.1 M HEPES buffer (Nippon Gene Co. Ltd.).

cDNA was synthesized using SuperScript IV Reverse Transcriptase (Thermo Fisher Scientific). The THUNDERBIRD™ Probe qRT-PCR kit (TOYOBO, Osaka, Japan) was used for qPCR analysis. The targets were amplified using CFX Connect (Bio-Rad, Hercules, CA, USA) at 95°C for 10 s (95°C for 5 s, 60°C for 20 s) × 50 cycles. Primers and probes used in this study are listed in Table 2.

**Table 2:** Sequences of primers and probes.

Primer or probe name	Sequence		
*HIV-1* forward	5′-TGT GTG CCC GTC TGT TGT GT-3′		
*HIV-1* reverse	5′-GCC GAG TCC TGC GTC GAG AG-3′		
*HIV-1* probe	5′-FAM-CAG TGG CGC CCG AAC AGG GA-BHQ1-3′		
*IPO8* forward	5′-CCA TCT GGC ATT AGG CAG CA-3′		
*IPO8* reverse	5′-GGG TTG TCC TTT TCC GTC CA-3′		
*IPO8* probe	5′-FAM-CCC TTT GAC CTT TGT CAC CCT GAA-BHQ1-3′		
*EZH1* forward	5′-CCT CAG TGC ACA CCC AAC AT-3′		
*EZH1* reverse	5′-TTA GGG GTG GCA TGA AAA GG-3′		
*EZH1* probe	5′-FAM-AGC AAT CTC TGC ACT CCT TCC ACA-BHQ1-3′		
*EZH2* forward	5′-GGA GTT TGC TGC TGC TCT CA-3′		
*EZH2* reverse	5′-TGC TGG GCC TGC TAC TGT TA-3′		
*EZH2* probe	5′-FAM-AAA CGT CCA GGA GGC CGC AGA A-BHQ1-3′		
*H1F0* forward	5′-GGC GGG CAG TGG ATA GTA AG-3′		
*H1F0* reverse	5′-AGC TCC CGG GTG TGA AAC TA-3′		
*H1F0* probe	5′-FAM-TCT GTG TGC ATG TGT GTG TTT GTG T-BHQ1-3′		

In ACH2 cells, cellular *HIV-1* expression was normalized by that of importin 8 (*IPO8*) (Ledderose et al., 2011). *HIV-1* expression in CD4^+^ T cells was calculated as *HIV-1* per HIV-1 provirus. The number of genomic *HIV-1* DNA (HIV provirus) copies was normalized by the number of genomic *IPO8* copies. Next, the normalized number of cellular *HIV-1* DNA copies was divided by the normalized HIV-1 provirus. Expression of H1 histone 1 family, member 0 (*H1F0*) was also normalized by that of *IPO8*.

### 2.6 Statistical analysis

qRT-PCR data in ACH2 cells sampled at 7 and 14 days were analyzed using the two-sample *t*-test (BellCurve for Excel ver. 4.07; Social Survey Research Information Co., Ltd., Tokyo, Japan). The variance of the population was estimated via the unbiased variance. The Kruskal–Wallis test was used to perform multiple comparisons of three or more experimental groups using the Bell Curve for Excel. The Steel–Dwass test was used to compare each experimental group individually. Differences were considered significant at *p* < 0.05.

### 2.7 Microarray transcriptome analysis

Total RNA was quantified using a Bioanalyzer 2,100 system (Agilent Technologies, Santa Clara, CA, USA) (Supplementary Figure 1). The Clariom™ S Assay for human samples (Thermo Fisher Scientific) was used for transcriptome analysis. Biotinylated sense-strand DNA targets were amplified from total RNA and hybridized using a GeneChip™ WT Plus Reagent Kit (Applied Biosystems™, Waltham, MA, USA) according to the manufacturer’s protocol. The cartridge was scanned using a GnenChip™ Scanner 3,000 7G system (Applied Biosistems™). Microarray images were analyzed using GeneChip™ Command Console Software ver. 3.2 and GeneChip™ Expression Console Software ver. 1.3.0. (Applied Biosistems™). Microarray data were analyzed using Transcriptome Analysis Console ver. 4.0 (Thermo Fisher Scientific). DAVID^1^ was used for the functional analysis of miRNAs (Sherman et al., 2022), Gene Ontology (GO) analysis of miRNA target mRNAs (Young et al., 2010), and Kyoto Encyclopedia of Genes and Genomes (KEGG) pathway enrichment analysis (Huang et al., 2009). The protein–protein interaction (PPI) network of proteins encoded by differentially expressed RNAs was analyzed using STRING^2^ (Szklarczyk et al., 2015). Hub genes in the completed PPI network were screened using CytoHubba Ver. 0.1 (Chin et al., 2014).

## 3 Results

### 3.1 Valemetostat effectively reactivates latently HIV-1-infected cells at longer treatment periods

**Figure 1:**
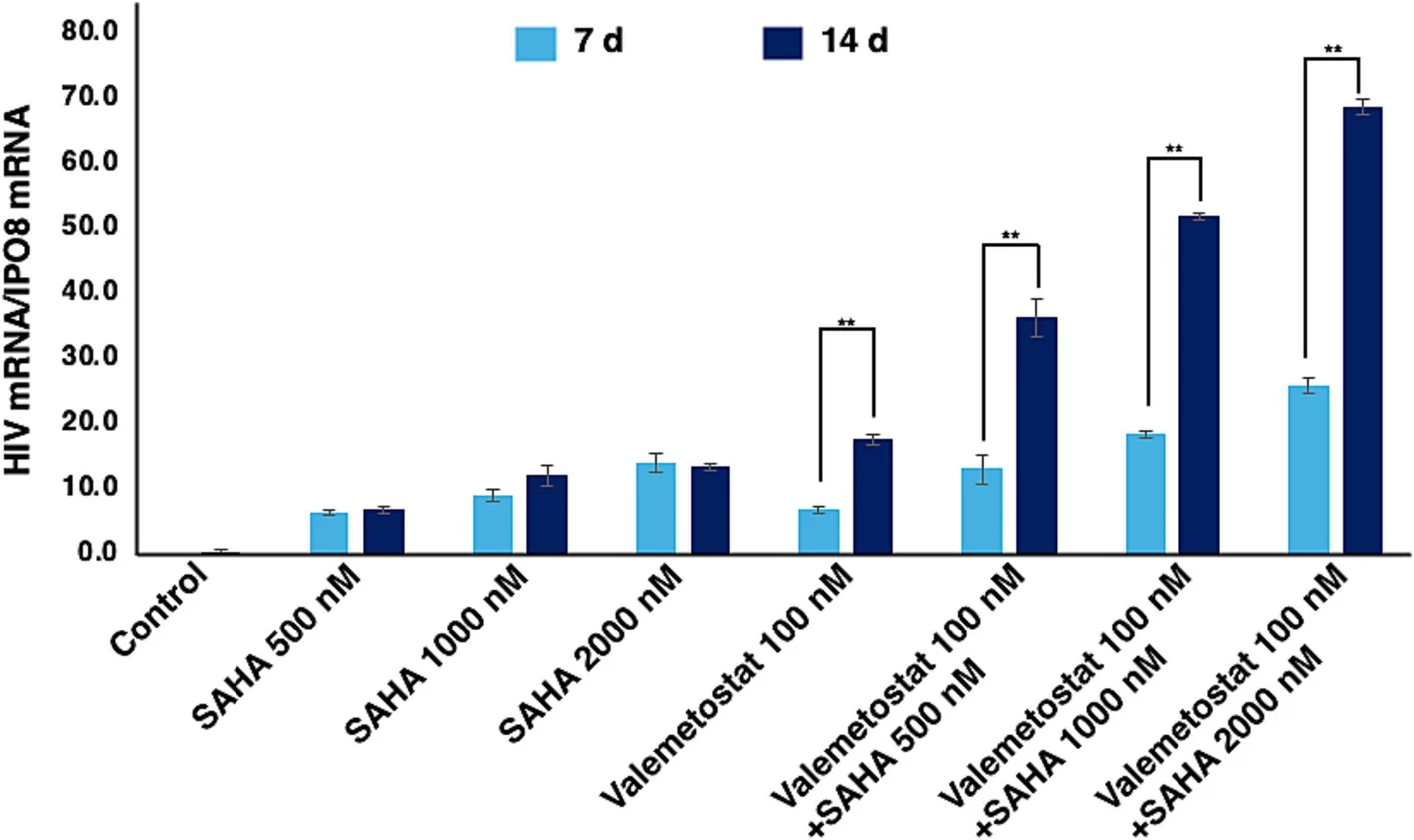
Reactivation of latently HIV-1-infected cells *in vitro* by valemetostat. qRT-PCR analysis of *HIV-1* mRNA expression in ACH2 cells that were treated with 100 nM valemetostat for 7–14 days; 100 nM, 500 nM, or 1 μM SAHA for 24 h; or a combination of valemetostat and SAHA with the latter being added 1 day before the last day of treatment with valemetostat.

We investigated the effects of 7–14-day treatment with valemetostat on the ACH2 cells, a model of latent HIV-1 infection (Ishida et al., 2006). The HDAC inhibitor SAHA (Matsuda et al., 2015) was used as the positive control. The qRT-PCR results showed that valemetostat reactivated cellular *HIV-1* expression in ACH2 cells, similar to the action of SAHA (Figure 1). A co-treatment of valemetostat and SAHA increased cellular *HIV-1* mRNA expression to a greater extent than treatment with either agent alone. In addition, the level of cellular *HIV-1* mRNA expression was higher in ACH2 cells treated with valemetostat for 14 days than in those treated for 7 days.

### 3.2 Maintenance of primary CD4^+^ T cells for >50 days *ex vivo*

**Figure 2:**
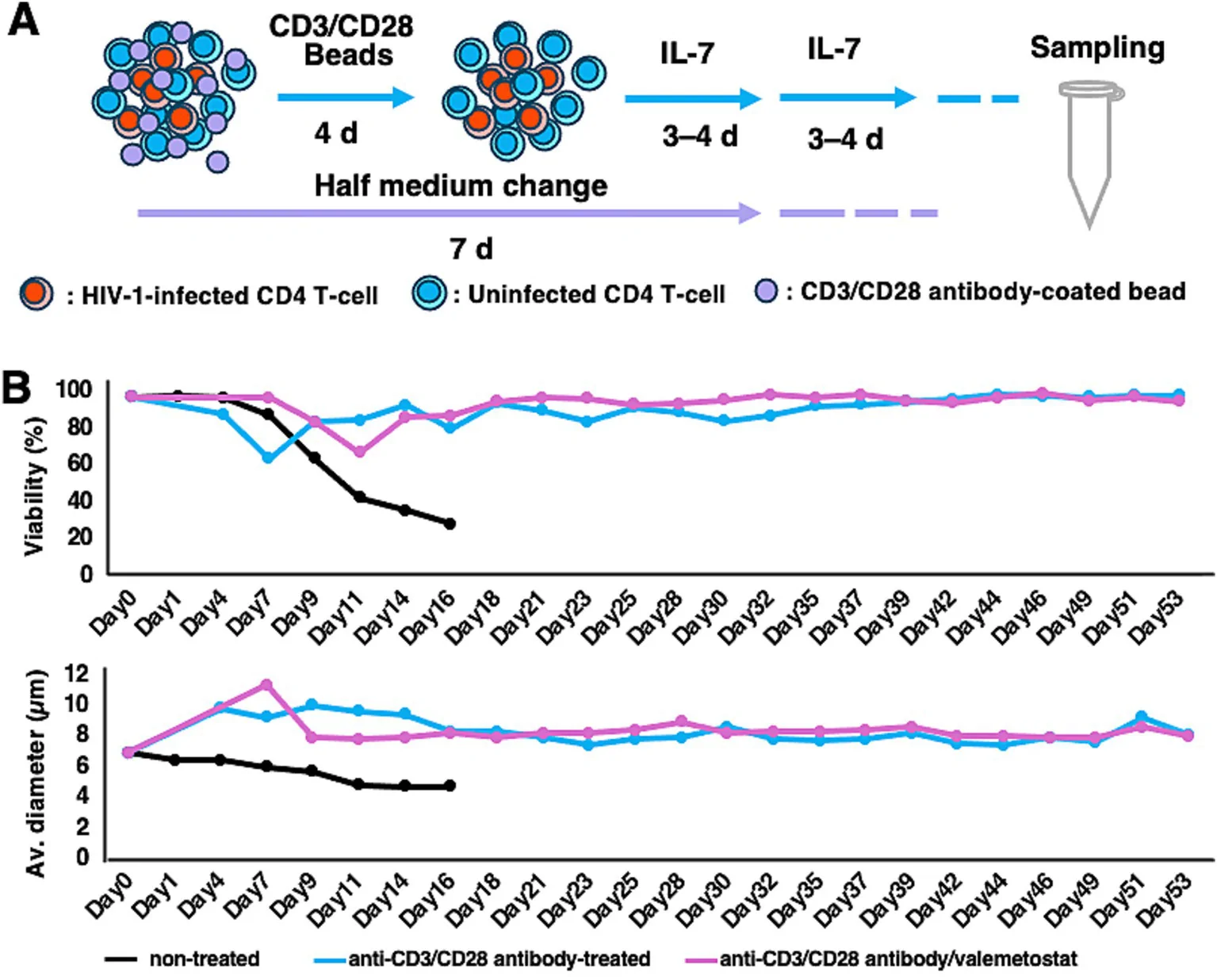
Maintenance of CD4^+^ T cells for more than 50 days *ex vivo*. **(A)** Long-term culture method for primary CD4^+^ T cells. Primary CD4^+^ T cells were treated with magnetic beads coated with anti-human CD3 and anti-human CD28 antibodies for 4 days and with IL-7 (5 ng/mL) every 3–4 days. Half of the medium was replaced with new culture medium every 7 days. **(B)** Viability and cell diameter of long-term cultured primary CD4^+^ T cells. These graphs show the results of one of the 12 cases. Black bar, non-treated CD4^+^ T cells; blue bar, CD4^+^ T cells treated with anti-human CD3 and anti-human CD28 antibodies; pink bar, CD4^+^ T cells treated with anti-human CD3 and anti-human CD28 antibodies and valemetostat.

Next, we investigated whether long-term treatment with valemetostat could efficiently induce cellular *HIV-1* expression in primary CD4^+^ T cells *ex vivo*. Although such cells cannot be maintained *ex vivo* for >7 days without immunostimulation, murine non-polarized primary CD4^+^ T cells can be maintained for a longer period following stimulation with anti-CD3/CD28 antibodies and IL-7 treatment (Kuzmichev et al., 2017; Murera et al., 2018). We successfully maintained primary CD4^+^ T cells derived from a patient with HIV-1 *ex vivo* for over 50 days (Figure 2A). These cells had high viability (over 80%) and normal cell diameter (over 7.5 μm) (Figure 2B), whereas primary CD4^+^ T cells cultured without beads coated with anti-CD3/CD28 antibodies had low viability (approximately 20%) and shrunken appearance (approximately 4–5 μm in diameter). The valemetostat-treated CD4^+^ T cells also maintained high viability for over 50 days, similar to that of the untreated CD4^+^ T cells.

### 3.3 Reactivation of latently HIV-1-infected cells ex vivo by valemetostat

**Figure 3:**
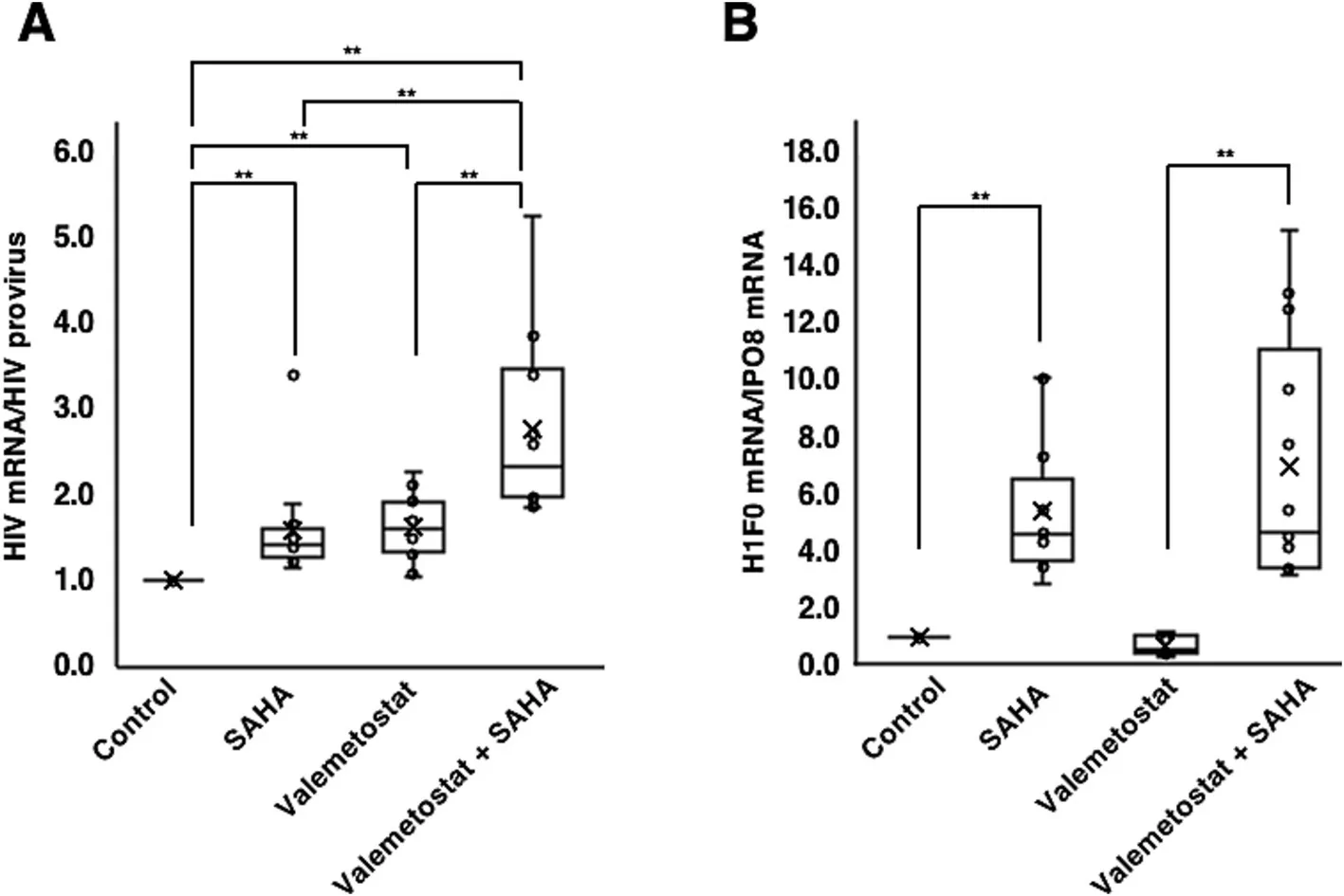
Reactivation by valemetostat of latently HIV-1-infected cells *ex vivo*. **(A)** qRT-PCR analysis of cellular *HIV-1* expression *ex vivo*. CD4^+^ T cells were treated with 1,000 nM valemetostat for 22 days and treated with 500 nM SAHA for 6 h on the last day of treatment with valemetostat (*n* = 12). **(B)** qRT-PCR analysis of the expression of SAHA-responsive gene *H1F0* (*n* = 12). **p < 0.01.

We investigated whether valemetostat acts as an LRA on CD4^+^ T cells derived from patients with HIV-1 (n = 12). CD4^+^ T cells were treated with 1 mM valemetostat for 22 days. SAHA (500 nM) was added for 6 h on day 22 of culture. qRT-PCR results showed that valemetostat reactivated latently HIV-1-infected cells *ex vivo* (Figures 3A, *P* < 0.05). The level of *HIV-1* mRNA expression induced by valemetostat was similar to that induced by SAHA (*p* > 0.05). When CD4^+^ T cells were treated with both valemetostat and SAHA, latently HIV-1-infected cells were more effectively reactivated than those treated with valemetostat alone (*p* < 0.05). Expression of the SAHA response gene *H1F0* was induced only in the SAHA-treated groups (Figures 3B, *P* < 0.05). *H1F0* expression was not induced by valemetostat, and the level of *H1F0* expression after co-treatment with valemetostat and SAHA was the same as that following treatment with SAHA alone (*p* > 0.05).

### 3.4 Comparison of HIV-1-reactivating potencies of valemetostat and EZH2-selective inhibitors GSK126 and E7438 *ex vivo*

**Figure 4:**
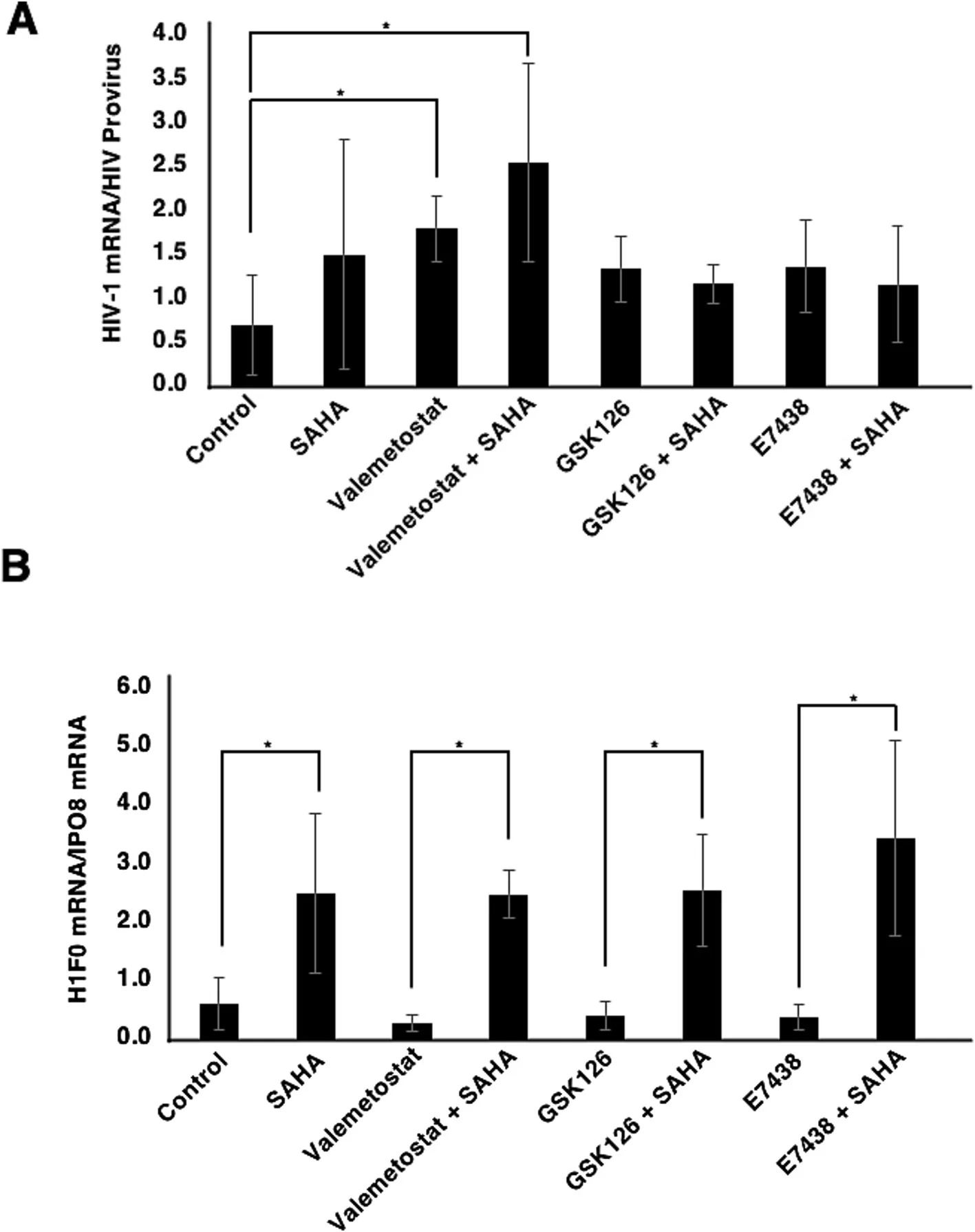
Comparison of the effects of valemetostat, GSK126, and E7538 on the reactivation of latently HIV-1-infected CD4^+^ T cells. **(A)** qRT-PCR analysis of cellular *HIV-1* mRNA expression *ex vivo*. CD4^+^ T cells were treated for 22 days with 1,000 nM valemetostat, 1,000 nM GSK126, or 1,000 nM E7438 alone or in combination with 500 nM SAHA added for 6 h on the last day of treatment with one of these drugs (*n* = 3). **(B)** qRT-PCR analysis of cellular *H1F0* expression (*n* = 3). *p < 0.05.

We investigated whether EZH1/2 dual inhibitor valemetostat could reverse HIV-1 latency *ex vivo* more effectively than EZH2-selective inhibitors GSK126 and E7438 (Duan et al., 2020). qRT-PCR revealed that valemetostat treatment induced *HIV-1* mRNA expression more effectively than treatment with GSK126 or E7438 (Figures 4A, *P* < 0.05). The effect of valemetostat on the activation of latently HIV-1-infected CD4^+^ T cells was increased by co-treatment with SAHA. Notably, the additive effect of co-treatment using SAHA with either GSK126 or E7438 was weaker than that of the combination of SAHA and valemetostat. *H1F0* expression was also induced by co-treatment with SAHA (Figures 4B, *P* < 0.05).

### 3.5 Cell membrane localization of protein products of 11 genes significantly upregulated by the treatment with valemetostat

**Figure 5:**
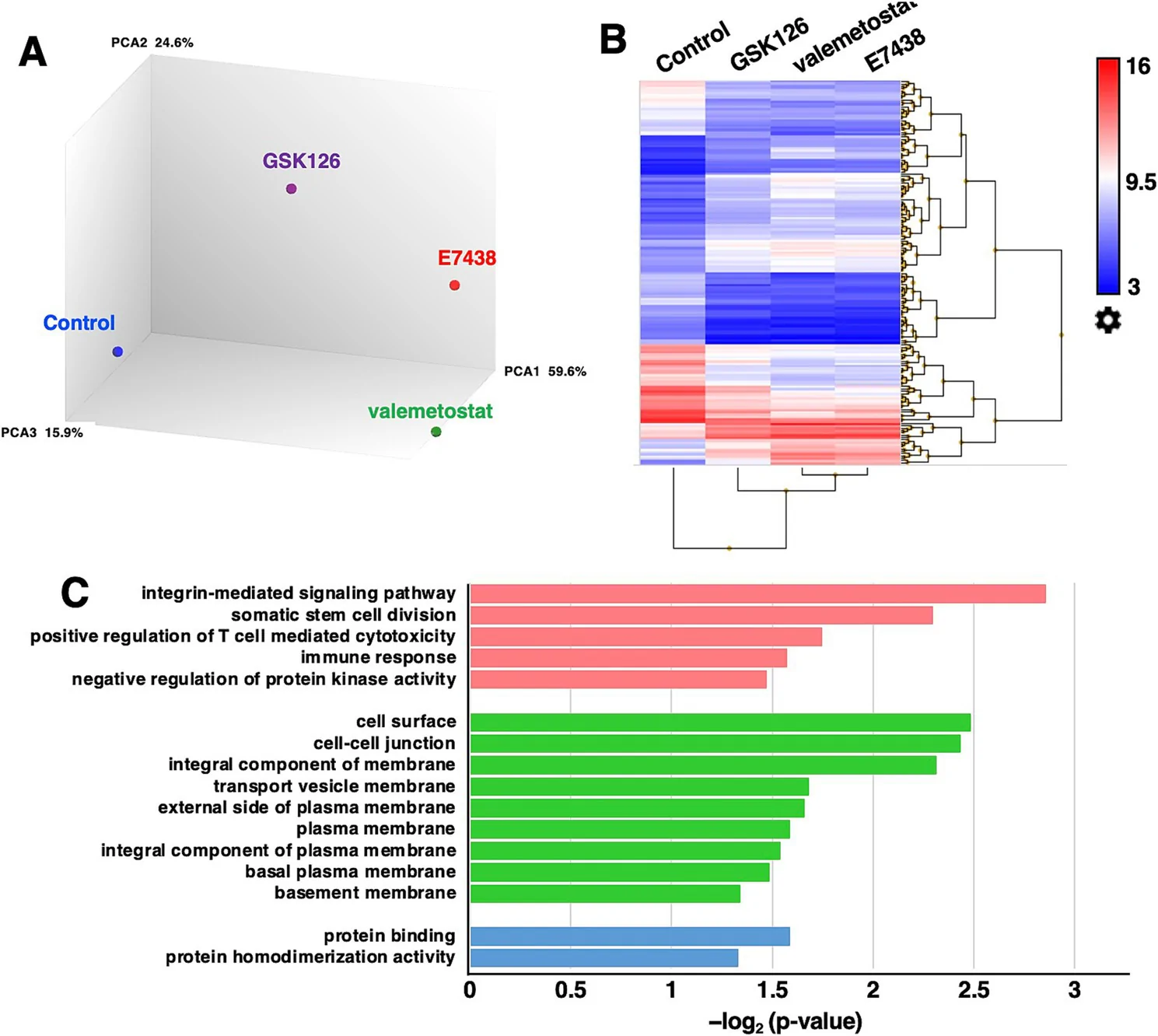
Microarray transcriptome analysis. **(A)** Principal component analysis of CD4^+^ T cells treated with 1,000 nM valemetostat, 1,000 nM GSK126, or 1,000 nM E7438 for 95 days (*n* = 1). Blue circle, control; purple circle, GSK126; green circle, valemetostat; red circle, E7438. The numbers on the right of PCA1, PCA2, and PCA3 indicate the contribution rate. **(B)** Heat map of genes whose expression levels changed more than threefold in CD4^+^ T cells treated with valemetostat, GSK126, or E7438 compared to the respective levels in untreated control cells. **(C)** Gene ontology analysis of the 11 genes that were three times more highly expressed in CD4^+^ T cells treated with valemetostat compared to their levels in GSK126- or E7438-treated cells. Pink, biological process; green, cellular component; blue, molecular function.

To evaluate the differences in the effects of valemetostat, GSK126, and E7438 on latently HIV-1-infected cells, the transcriptomes of CD4^+^ T cells were analyzed upon treatment with drugs for 95 days. The quality of the extracted total RNA was examined (Supplementary Figure 1), and the transcriptomes were analyzed using a microarray. To investigate the similarity of the transcriptomes between each sample, we performed principal component analysis on each transcriptome. The principal component analysis plot shown in Figure 5A indicates that the closer the samples are plotted, the greater the similarity between each sample, and it visually shows the overall relationship between samples. When the gene expression patterns after valemetostat treatment were compared with the gene expression patterns after E7438 and GSK126 treatment, the gene expression patterns after valemetostat treatment were more similar to the gene expression patterns after E7438 treatment than to the gene expression patterns after GSK126 treatment (Figure 5A). Figure 5B shows a heat map of genes whose expression levels changed >3-fold after treatment with valemetostat, GSK126, or E7438 (Supplementary Table S1). Expression levels of 177 genes increased >3-fold after valemetostat treatment compared with the effect of GSK126 (Supplementary Table S2). Similarly, for 17 genes, expression levels increased by >3-fold in cells treated with valemetostat compared to those in cells treated with E7438 (Supplementary Table S3). Among the genes whose expression levels increased >3-fold after treatment with valemetostat, 11 genes were common to both GSK126 and E7438. GO analysis of these 11 genes revealed that their protein products localize to the cell membrane and are involved in integrin-mediated signal transduction, cell division, and immune responses (Figure 5C and Table 3). In a similar comparison, only two genes showed a > 3-fold decrease in expression (Supplementary Table 4).

**Table 3:** Eleven genes with a 3-fold higher expression in valemetostat-treated CD4^+^ T cells than in GSK126- or E7438-treated cells.

Gene symbol	Description	Valemetostat average (log_2_)	GSK126 average (log_2_)	E7438 average (log_2_)	Fold change valemetostat vs. GSK126	Fold change valemetostat vs. E7438
*CDKN2A*	Cyclin-dependent kinase inhibitor 2A	11.71	5.08	9.17	98.96	5.8
*KIT*	v-kit Hardy–Zuckerman 4 feline sarcoma viral oncogene homolog	6.8	4.57	4.57	4.7	4.7
*HLA-DRA*	Major histocompatibility complex, class II, DR alpha	10.63	7.66	8.58	7.87	4.15
*ADAMTS1*	ADAM metallopeptidase with thrombospondin type 1 motif 1	9.25	7	7.31	4.76	3.84
*CEACAM1*	Carcinoembryonic antigen-related cell adhesion molecule 1 (biliary glycoprotein)	10.94	7.55	9.04	10.48	3.74
*ULBP1*	UL16 binding protein 1	6.77	4.25	4.93	5.75	3.58
*SLC4A4*	Solute carrier family 4 (sodium bicarbonate cotransporter), member 4	9.2	4.9	7.43	19.75	3.42
*SLC35F3*	Solute carrier family 35, member F3	10.59	10.59	8.86	8.68	3.32
*CD80*	CD80 molecule	9.65	7.31	7.94	5.08	3.28
*DST*	Dystonin	10.01	5.58	8.3	21.55	3.27
*STX3*	Syntaxin 3	12.94	9.84	11.35	8.56	3.02

### 3.6 Protein products of 21 hub genes are related to cytokine receptor interactions and JAK–STAT signaling

**Figure 6:**
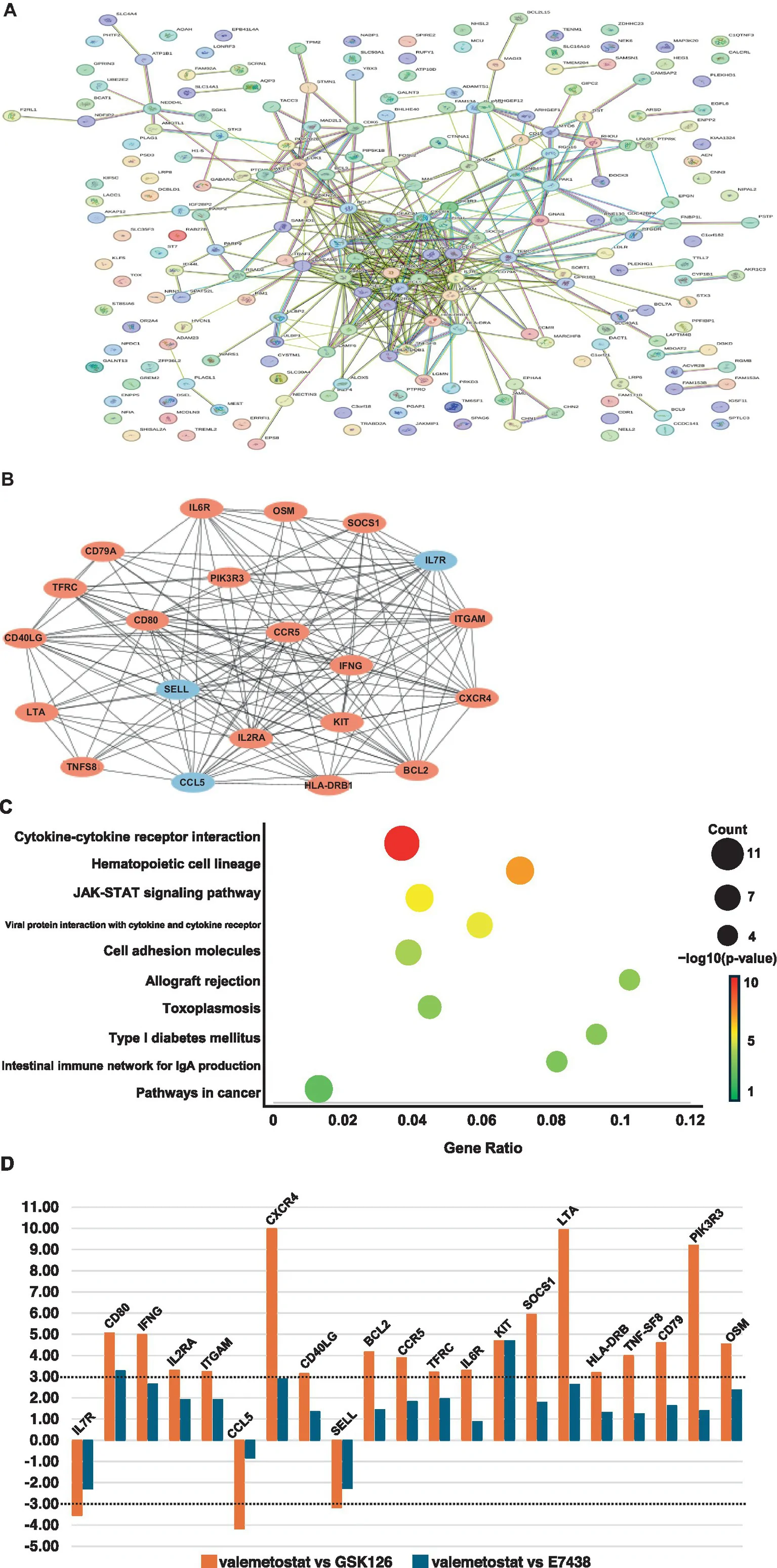
Analysis of genes with more than threefold changes in expression levels following treatment with valemetostat compared to the changes caused by the treatment with the EZH2-selective inhibitor GSK126. **(A)** PPI network of 227 genes whose expression levels changed more than threefold in CD4^+^ T cells upon treatment with valemetostat, compared to the changes caused by the treatment with GSK126. The colors of the edge lines indicate the following: light blue, database-supported associations; purple, experimentally demonstrated associations; red, fusion genes; yellow–green, associations supported by previous studies; green, gene neighborhoods; blue, cooccurring genes; black, coexpressing genes. **(B)** A network of 21 hub genes extracted from the 227 genes whose expression levels changed in valemetostat-treated cells, compared to their expression in GSK126-treated cells. The color of the nodes indicates that red indicates genes that were highly expressed in valemetostat, and blue indicates genes that were expressed at low levels in valemetostat. **(C)** KEGG enrichment pathway analysis of the 21 hub genes. **(D)** Twenty-one hub genes with more than threefold changes in expression levels changed in valemetostat-treated cells compared to their expression levels in GSK126- and E7438-treated cells. The dotted lines show positive and negative threefold change levels.

Transcriptomic analysis showed that expression levels of 227 genes in valemetostat-treated cells changed compared with those in GSK126-treated CD4^+^ T cells (3-fold change, Supplementary Table 5). The PPI network revealed that proteins encoded by these 227 genes formed 212 nodes, 68 isolates, and 387 edges (Figure 6A and Supplementary Table 5). The 21 hub genes were identified from the PPI network (Table 4). The network of these 21 hub genes is shown in Figure 6B (Supplementary Table 6). GO analysis revealed that protein products of the 21 hub genes were localized in the cell membrane, cytoplasmic vesicles, and extracellular space (Supplementary Figure 2). The 21 hub genes were involved in immune response, T-cell proliferation, and B-cell differentiation. KEGG enrichment pathway analysis showed that the 21 hub genes contributed remarkably to the cytokine–cytokine receptor interactions and were involved in the JAK–STAT signaling pathway (Figure 6C). The same analysis, including PPI network analysis, was conducted for valemetostat and E7438, but only seven genes had expression levels altered by >3-fold (Supplementary Figure 3 and Supplementary Table 7). Expression levels of the 21 hub genes extracted from the transcriptomes modulated by valemetostat and GSK126 were also examined in the E7438-modulated transcriptome (Figure 6D). Expression levels of the 21 hub genes in the analysis of valemetostat vs. E7438 did not increase or decrease by >3-fold; however, they showed a similar increase/decrease pattern as observed in the comparison of valemetostat with GSK126.

**Table 4:** Twenty-one hub genes extracted from the list of 227 genes exhibiting a > 3-fold change in expression levels in valemetostat-treated CD4^+^ T cells.

Gene symbol	Description	Valemetostat (log_2_)	GSK126 (log_2_)	E7438 (log_2_)	Fold change valemetostat vs. GSK126	Fold change valemetostat vs. E7438	References
*IL7R*	Interleukin 7 receptor	14.94	16.76	16.13	−3.53	−2.28	Goonetilleke et al. (2019) and Winer et al. (2022)
*CD80*	CD80 molecule	9.65	7.31	7.94	5.06	3.27	Carbone et al. (2024)
*IFNG*	Interferon, gamma	5.66	3.34	4.26	4.99	2.64	Stadhouders et al. (2018) and Qi et al. (2021)
*IL2RA*	Interleukin 2 receptor, alpha	12.9	11.18	11.96	3.29	1.92	Li et al. (2022) and Zhu et al. (2020)
*ITGAM*	Integrin, alpha M (complement component 3 receptor 3 subunit)	7.93	6.24	6.99	3.23	1.92	Villanueva et al. (2022)
*CCL5*	Chemokine (C-C motif) ligand 5	10.02	12.08	9.74	−4.17	−0.82	Mukaida et al. (2020)
*CXCR4*	Chemokine (C-X-C motif) receptor 4	14.12	10.8	12.59	9.99	2.89	Shimba and Ikuta (2020)
*CD40LG*	CD40 ligand	9.62	7.97	9.2	3.14	1.34	Hale and Ahmed (2015)
*SELL*	Selectin L	12.88	14.54	14.06	−3.16	−2.27	Caccamo et al. (2018)
*BCL2*	B-cell CLL/lymphoma 2	12.68	10.62	12.16	4.17	1.43	Chetoui et al. (2010)
*CCR5*	Chemokine (C-C motif) receptor 5 (gene/pseudogene)	5.21	3.25	4.34	3.89	1.83	Wang et al. (2024)
*TFRC*	Transferrin receptor	10.37	8.69	9.4	3.20	1.96	Voss et al. (2023)
*IL6R*	Interleukin 6 receptor	8.5	6.78	8.71	3.29	0.86	Prado et al. (2021)
*KIT*	v-kit Hardy–Zuckerman 4 feline sarcoma viral oncogene homolog	6.8	4.57	4.57	4.69	4.69	Marega et al. (2021)
*SOCS1*	Suppressor of cytokine signaling 1	8.09	5.52	7.25	5.94	1.79	Chandrakar et al. (2020)
*LTA*	Lymphotoxin alpha	12.39	9.08	11	9.92	2.62	Sailliet et al. (2023)
*HLA-DRB*	Major histocompatibility complex, class II, DR beta 1	10.27	8.6	9.88	3.18	1.31	Tippalagama et al. (2021)
*TNF-SF8*	Tumor necrosis factor (ligand) superfamily, member 8	12.17	10.17	11.86	4.00	1.24	Sun et al. (2024)
CD79	CD79a molecule, immunoglobulin-associated alpha	10.12	7.92	9.42	4.59	1.62	Luger et al. (2013)
PIK3R3	Phosphoinositide-3-kinase, regulatory subunit 3 (gamma)	7.46	4.26	6.99	9.19	1.39	Murter and Kane (2020)
OSM	Oncostatin M	9.01	6.83	7.77	4.53	2.36	Han et al. (2023)

## 4 Discussion

In this study, we demonstrated that the EZH1/2 dual inhibitor valemetostat demonstrated higher efficacy in reactivating latently HIV-1-infected cells than the EZH2 selective inhibitors GSK126 and E7438. Valemetostat was more effective in reactivating latently HIV-1-infected cells upon treatment over a longer period or in combination with SAHA. Our results support those of previous studies that co-treatment with an EZH2-selective inhibitor and SAHA enhanced cellular *HIV-1* expression stronger than either agent alone (Tripathy et al., 2015). In addition, in the present study, we demonstrated that SAHA enhanced valemetostat-induced reactivation of latent HIV-1 infection *ex vivo*. However, the expression of the SAHA-responsive gene *H1F0* was not enhanced by valemetostat, suggesting that this drug regulates *HIV-1* expression by a mechanism not involving *H1F0* upregulation. In future experiments, a detailed analysis of epigenetic changes caused by valemetostat may be warranted.

Valemetostat afforded similar reactivation of latently HIV-1-infected cells compared with GSK126 and E7438, although valemetostat tended to induce higher *HIV-1* expression than GSK126 and E7438. Yamagishi et al. (2019) reported that EZH1 and EZH2 play complementary roles in regulating gene expression and that each regulates the expression of their own target genes, suggesting that the inhibition of EZH2 alone may not be sufficient (Yamagishi et al., 2019). Although the similarity between valemetostat and E7438 was high in the transcriptome analysis, valemetostat induced stronger gene expression changes compared to E7438. This is thought to be because valemetostat can inhibit EZH1 and EZH2 simultaneously and therefore has a greater effect than E7438 does. For this reason, it is thought that valemetostat shows a higher HIV-1 reactivation efficiency than E7438. SAHA more strongly enhanced the action of valemetostat compared with GSK126 and E7438. The induction of the SAHA-responsive gene *H1F0* expression confirmed the efficacy of SAHA treatment. The additive effects of GSK126 and E7438 when co-treated with SAHA will be a topic for future studies.

The similarity of transcription profiles of valemetostat- and E7438-treated cells precluded comparison of these two drugs. In contrast, transcriptomes of valemetostat and GSK126 were different, and 21 distinct hub genes were identified. As valemetostat plays a role in regulating transcription, we expected that the genes with expression levels regulated by valemetostat would primarily consist of transcription factors. However, protein products of the genes that were differentially regulated by valemetostat and GSK126 were involved in the signal transduction systems localized on the cell membrane. The mechanism by which changes in the expression of these genes mediate the effects of valemetostat remains unclear. To elucidate the efficacy of valemetostat, it will be essential to investigate the relationships between these 21 genes.

This study has some limitations. The transcriptome analysis showed that valemetostat increased the expression of genes related to the JAK–STAT signaling pathway. In contrast, as the proportion of HIV-infected CD4^+^ T cells isolated from HIV-infected individuals is low (Woldemeskel et al., 2020), it was difficult to extract only HIV-1-infected cells and establish the mechanism of valemetostat action precisely. To solve this problem, it may be necessary to examine the direct effect of valemetostat on HIV-infected cells using *in vitro* experiments in cell lines, such as ACH2, which was used in this study.

## 5 Conclusion

The EZH1/2 dual inhibitor valemetostat reactivated latently HIV-l-infected cells *ex vivo* and tended to induce cellular *HIV-1* mRNA expression more effectively than the existing drugs, EZH2-selective inhibitors GSK126 and E7438. A co-treatment with SAHA and valemetostat generated a stable additive effect that surpassed the effects of the combined treatment of SAHA with GSK126 or E7438. The transcriptomes of valemetostat- and E7438-treated cells were highly similar but differed from the transcriptome of GSK126-treated cells. We identified 21 hub genes that showed marked increases in expression levels upon valemetostat treatment. The products of these 21 genes are located on the cell membrane and involved in immune responses. This study provides new information relevant to the development of future treatments that would effectively reactivate latently HIV-1-infected cells and eliminate HIV-1-infected cells from patients.

## Data Availability

All data supporting this study are available in the article 502 and supplementary data. The microarray data analyzed in this study are available in the DDBJ Genomic Expression Archive (GEA accession number : E-GEAD-876, https://ddbj.nig.ac.jp/public/ddbj_database/gea/experiment/E-GEAD-000/EGEAD-876/) and NCBI database (BioProject accession number : PRJDB19056, https://www.ncbi.nlm.nih.gov/bioproject/?term=PRJDB19056).

## Footnotes

^1^ https://david.ncifcrf.gov/

^2^ https://string-db.org/
