# Correction: Enhancer of zeste homolog 1/2 dual inhibitor valemetostat outperforms enhancer of zeste homolog 2-selective inhibitors in reactivating latent HIV-1 reservoirs *ex vivo*

**DOI:** 10.3389/fmicb.2026.2009976

**Published:** 2026-10-30

**Authors:** Ayako Sedohara, Tomohiko Koibuchi, Makoto Yamagishi, Michiko Koga, Kotaro Arizono, Kazuhiko Ikeuchi, Tadashi Kikuchi, Makoto Saito, Eisuke Adachi, Takeya Tsutsumi, Daisuke Honma, Kazushi Araki, Kaoru Uchimaru, Hiroshi Yotsuyanagi

**Affiliations:** 1 Division of Infectious Diseases, Advanced Clinical Research Center, Institute of Medical Science, The University of Tokyo, Minato-ku, Tokyo, Japan; 2 Department of Infectious Disease and Applied Immunology, IMSUT Hospital of The Institute of Medical Science, The University of Tokyo, Minato-ku, Tokyo, Japan; 3 Department of Computational Biology and Medical Sciences, Graduate School of Frontier Sciences, The University of Tokyo, Bunkyo-ku, Tokyo, Japan; 4 Department of Infectious Diseases, Faculty of Medicine, The University of Tokyo, Bunkyo-ku, Tokyo, Japan; 5 Modality Research Laboratories III, Daiichi Sankyo Co., Ltd., Shinagawa-ku, Tokyo, Japan; 6 Early Clinical Development Department, Daiichi Sankyo Co., Ltd., Shinagawa-ku, Tokyo, Japan

**Keywords:** HIV-1 reservoir, latency reversing agents, EZH1/2 dual inhibitor, valemetostat, suberoylanilide hydroxamic acid, GSK126, E7438

There was a mistake in the caption of **Figure 2** as published. In the legend to **Figure 2**, “IL-7 (5 μg/mL)” should read “IL-7 (5 ng/mL).” The corrected caption of **Figure 2** appears below.

“Maintenance of CD4+ T cells for more than 50 days *ex vivo*. **(A)** Long-term culture method for primary CD4+ T cells. Primary CD4+ T cells were treated with magnetic beads coated with anti-human CD3 and anti-human CD28 antibodies for 4 days and with IL-7 (5 ng/mL) every 3–4 days. Half of the medium was replaced with new culture medium every 7 days. **(B)** Viability and cell diameter of long-term cultured primary CD4+ T cells. These graphs show the results of one of the 12 cases. Black bar, non-treated CD4+ T cells; blue bar, CD4+ T cells treated with anti-human CD3 and anti-human CD28 antibodies; pink bar, CD4+ T cells treated with anti-human CD3 and anti-human CD28 antibodies and valemetostat.”

“After the removal of the beads, 5 mg/mL interleukin 7 (IL-7) was added every 3–4 days (Murera et al., 2018).” should be: “After the removal of the beads, 5 ng/mL interleukin 7 (IL-7) was added every 3–4 days (Murera et al., 2018).”

A correction has been made to the section **2 Materials and methods**, *2.3 Long-term primary CD4*^+^ *T cell culture*, Paragraph Number 1:

“Peripheral blood mononuclear cells were isolated using SepMate™ (STEMCELL Technologies, Vancouver, BC, Canada) according to the manufacturer's protocol. CD4^+^ T cells were isolated by negative selection using an EasySep™ Human CD4^+^ T Cell Isolation Kit (STEMCELL Technologies) and cultured at 37 °C in the atmosphere of 95% air and 5% CO_2_ in a 48-well flat-bottomed plate (Sumitomo Bakelite Co.) filled with 500 μL of RPMI-1640 medium (plus L-glutamine, without phenol red; Gibco) supplemented with 10% 0.22 μM filtered fetal bovine serum (Thermo Fisher Scientific), 0.6% of penicillin–streptomycin solution (Merck), and 300 nM efavirenz (Merck), which prevented new HIV-1 infection. To maintain primary CD4^+^ T cells *ex vivo* for >7 days, they were treated with beads coated with antibodies against CD3 and CD28 (STEMCELL Technologies) for 4 days (Kuzmichev et al., 2017). After the removal of the beads, 5 ng/mL interleukin 7 (IL-7) was added every 3–4 days (Murera et al., 2018). Half of the culture medium was renewed every 7 days.”

The original version of this article has been updated.

