# Supplementary material for: Enhancer of zeste homolog 1/2 dual inhibitor valemetostat outperforms enhancer of zeste homolog 2-selective inhibitors in reactivating latent HIV-1 reservoirs ex vivo
Source: Front Microbiol. 2025 Apr 10;16:1581330. doi: 10.3389/fmicb.2025.1581330 (PMC12020917; doi:10.3389/fmicb.2025.1581330)
Supplement: Supplementary file 1 [file Data_Sheet_1.docx]

Supplementary Material

# Supplementary Data

Supplementary Material should be uploaded separately on submission. Please include any supplementary data, figures and/or tables.

Supplementary material is not typeset so please ensure that all information is clearly presented, the appropriate caption is included in the file and not in the manuscript, and that the style conforms to the rest of the article.

# Supplementary Figures and Tables

For more information on Supplementary Material and for details on the different file types accepted, please see [here](https://www.frontiersin.org/guidelines/author-guidelines#supplementary-material).

## Supplementary Figures


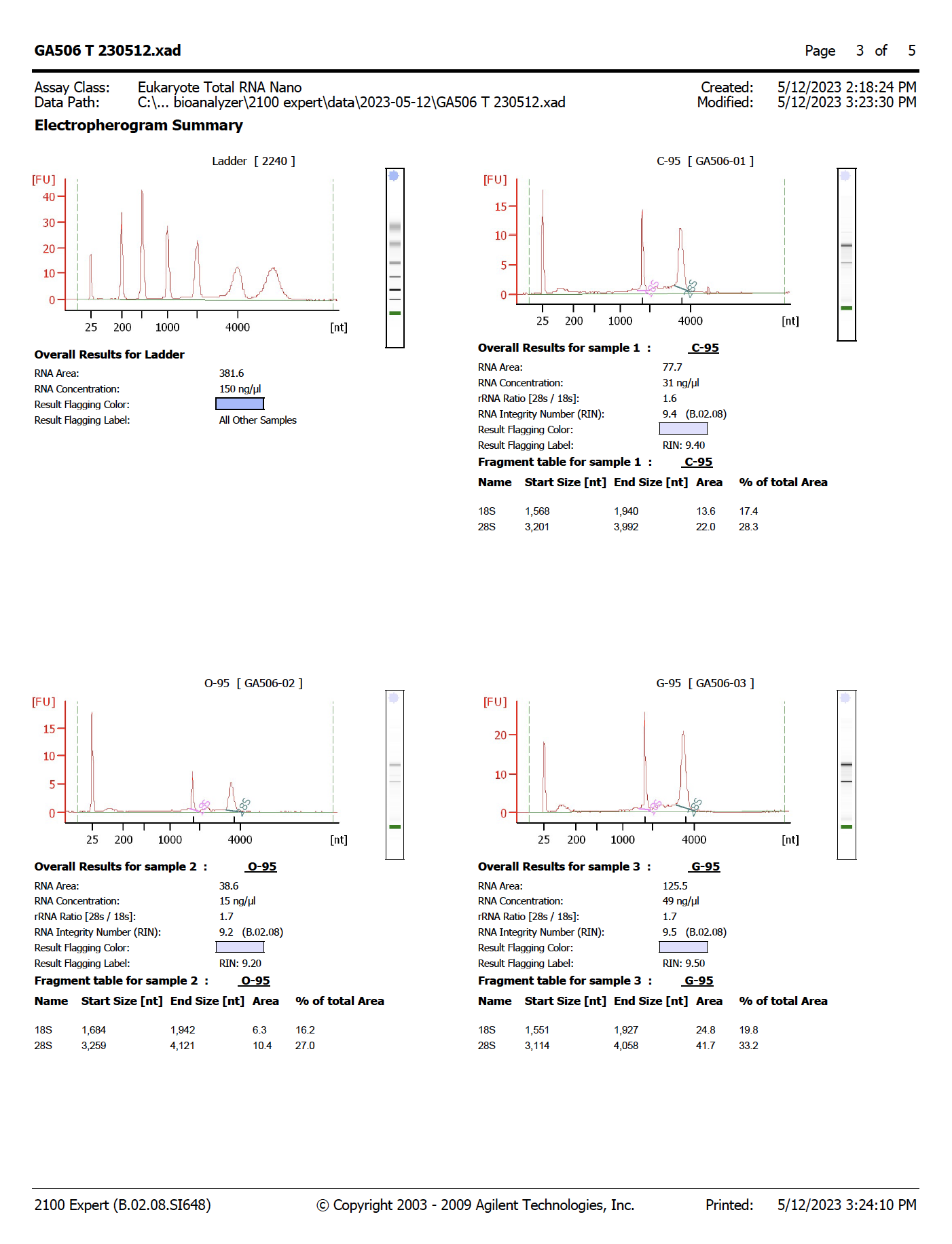

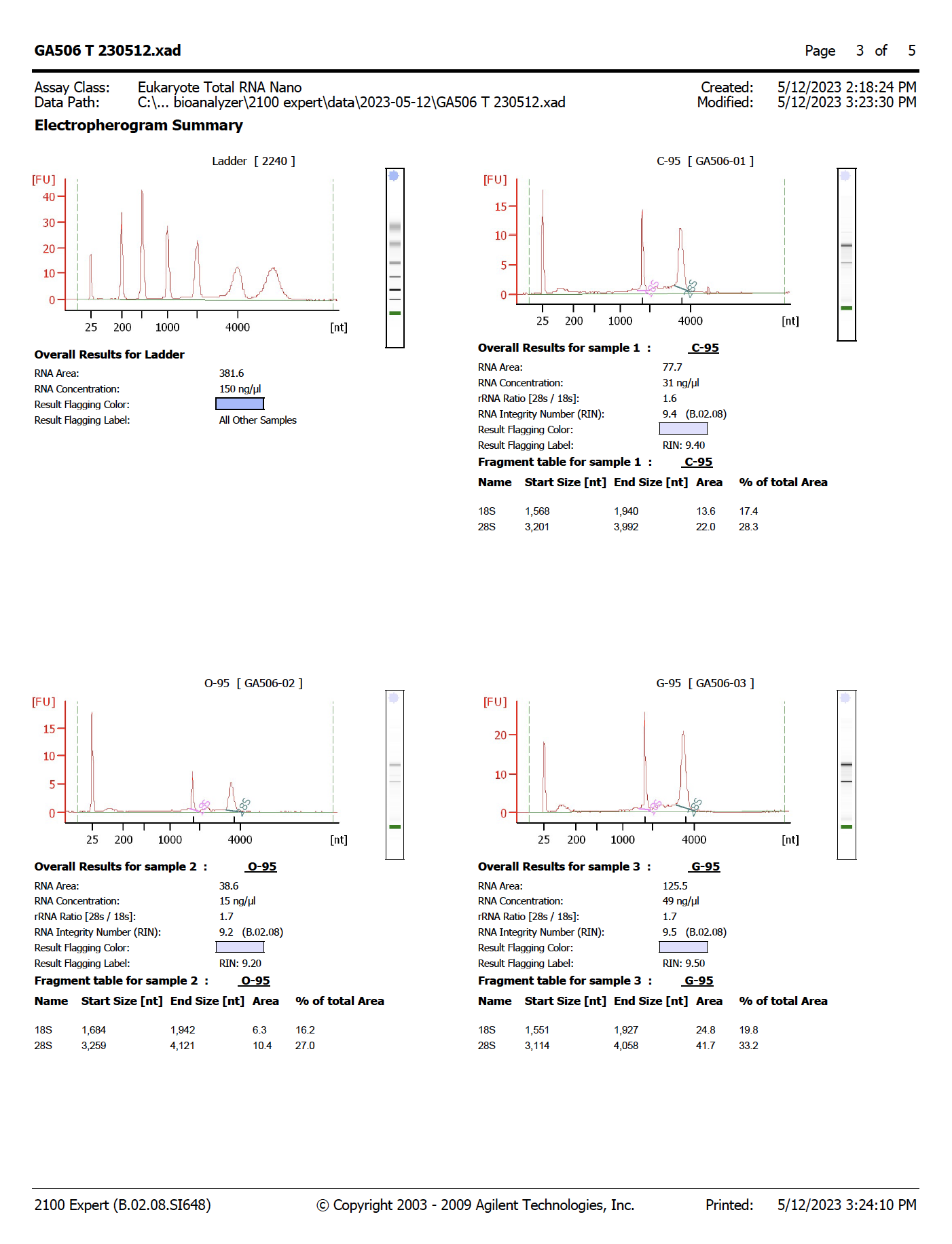

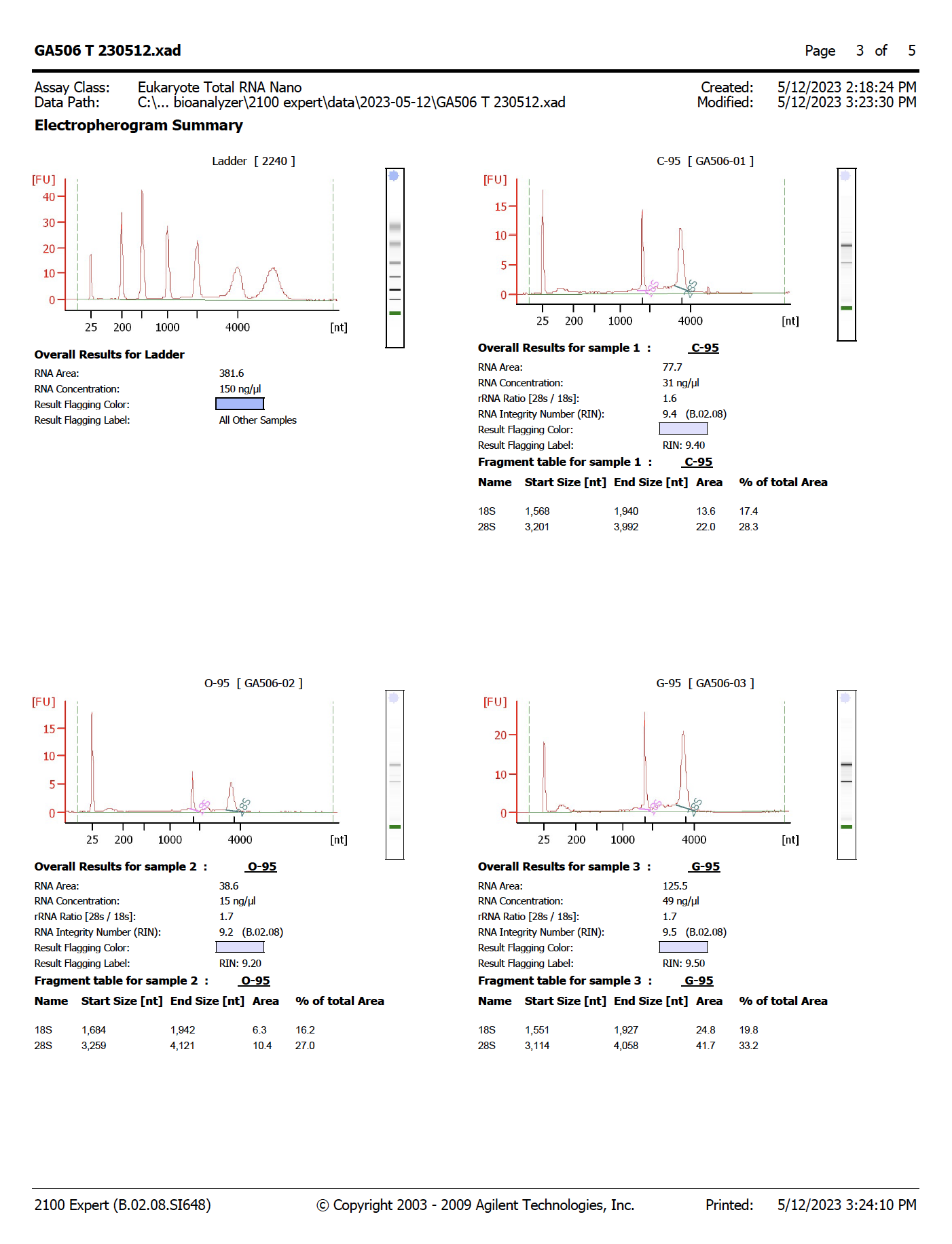

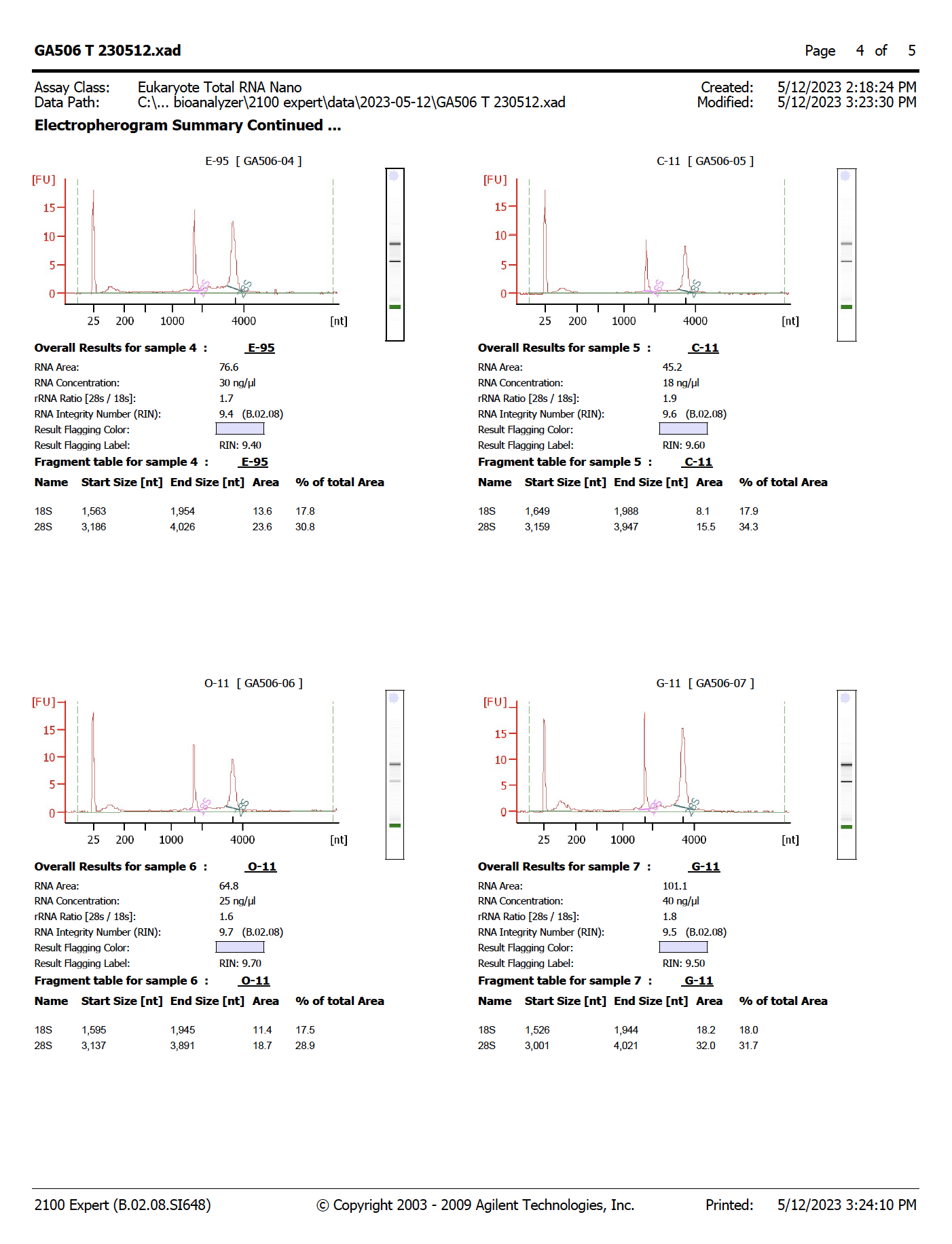


**A**

**B**

**C**

**D**

**valemetostat**

**control**

**control**

**valemetostat**

**E7438**

**GSK126**

**E7438**

**GSK126**

**Supplementary Figure 1.** Total RNA quantification using a Bioanalyzer 2100 system.

**
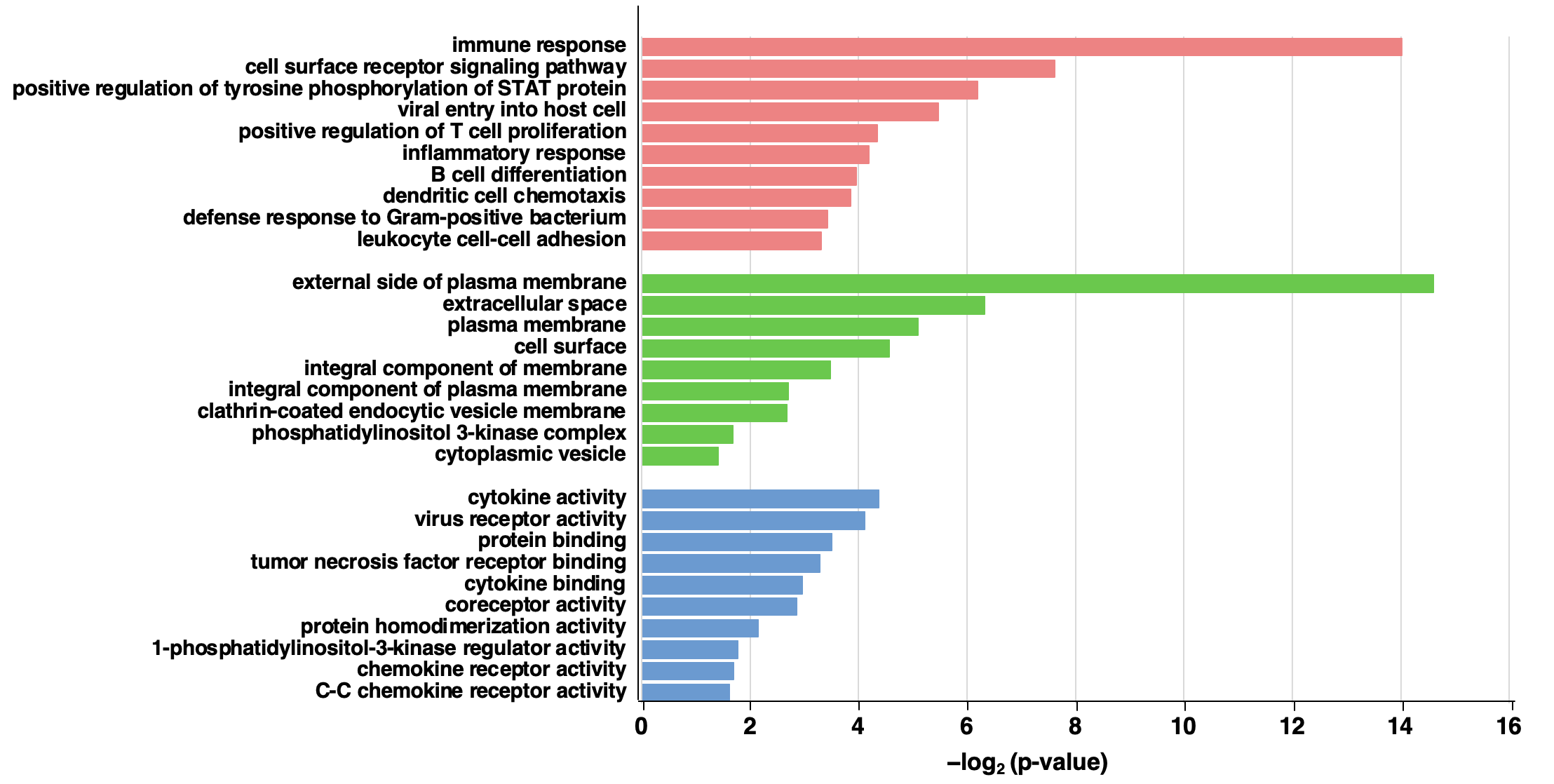
**

**Supplementary Figure 2. Gene ontology analysis of 21 hub genes.** Twenty-one hub genes were extracted from the list of 227 genes whose expression levels changed in CD4+ T cells upon treatment with valemetostat compared to their levels in the GSK126-treated cells. Pink, biological process; green, cellular component; blue, molecular function.


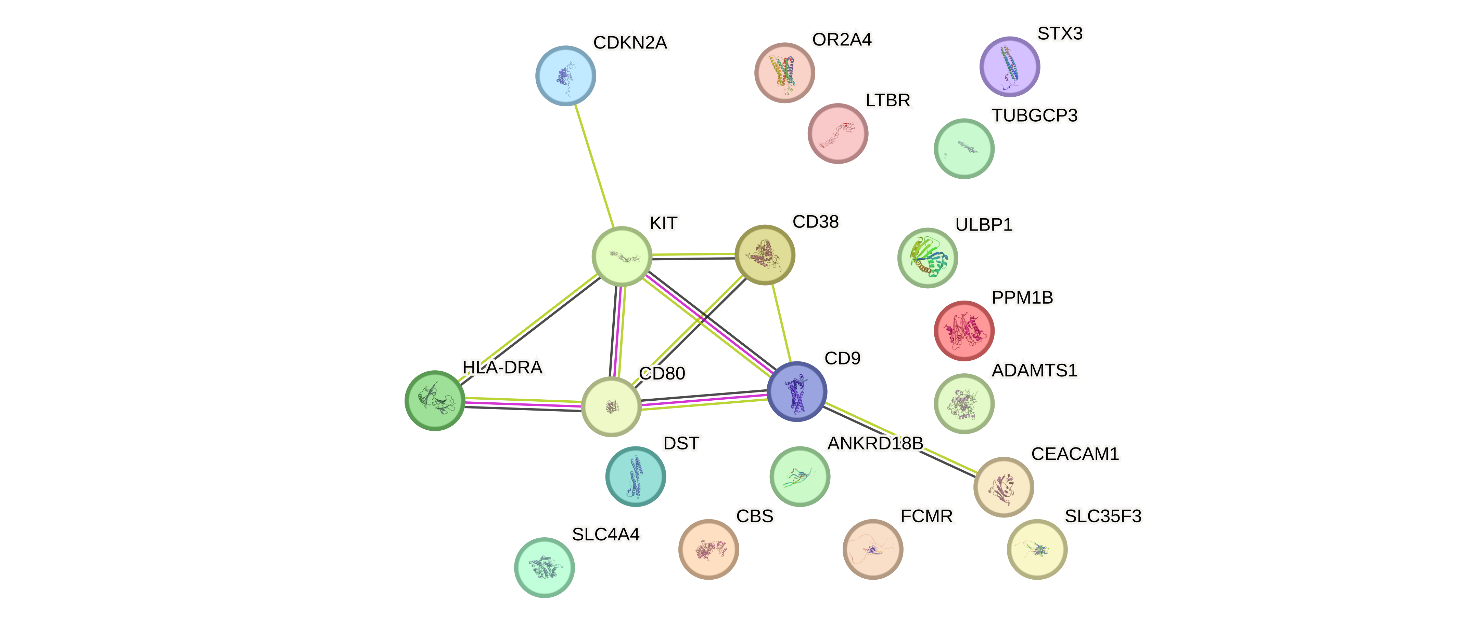


**A**


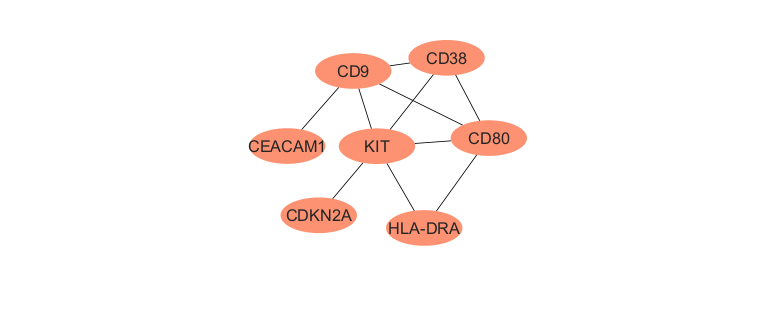


**B**

**Supplementary Figure 3.** PPI and hub network of seven genes whose expression levels changed more than threefold in valemetostat-treated CD4+ T cells compared to their levels in E7438-treated cells. (A) PPI network. (B) The network of seven hub genes.

## Supplementary Tables

Supplementary Tables are extremely lengthy, they have been submitted as a separate Excel file.
